# Inflammatory and Oxidative Stress Responses to High-Carbohydrate and High-Fat Meals in Healthy Humans

**DOI:** 10.1155/2012/238056

**Published:** 2012-02-13

**Authors:** S. Gregersen, D. Samocha-Bonet, L. K. Heilbronn, L. V. Campbell

**Affiliations:** ^1^Department of Endocrinology and Metabolism, Aarhus University Hospital, Tage-Hansensgade 2, 8000 Aarhus C, Denmark; ^2^Diabetes and Obesity Program, Garvan Institute of Medical Research, 384 Victoria Street, Darlinghurst, NSW 2010, Australia; ^3^Discipline of Medicine, The University of Adelaide, Eleanor Harrald Building, Frome Road, Adelaide, SA 5005, Australia

## Abstract

The postprandial state is hypothesised to be proinflammatory and prooxidative, but the relative contributions of fat versus carbohydrate are unclear. Therefore, we examined inflammation and oxidative stress responses in serum and skeletal muscle before and after 1000 kcal meals, which were high in either fat or carbohydrate in 15 healthy individuals. Serum and muscle expression of IL6 was elevated 3 hours after each meal, independently of macronutrient composition (*P* < 0.01). Serum IL18 was decreased after high-fat meal only (*P* < 0.01). Plasma total antioxidative status and muscle Cu/Zn-superoxide dismutase were decreased after high-carbohydrate meal only (*P* < 0.05). We conclude that a high-carbohydrate meal may evoke a greater postprandial oxidative stress response, whereas both fat and carbohydrate increased IL6. We speculate that the observed increases in postprandial IL6, without increases in any other markers of inflammation, may indicate a normal IL6 response to enhance glucose uptake, similar to its role postexercise.

## 1. Introduction

Inflammation and oxidative stress are postulated to impair beta-cell function and exacerbate insulin resistance in type 2 diabetes [1, 2] and often coexist in atherosclerosis and cardiovascular disease [3]. Many studies have reported inflammation and oxidative stress in the fasting state in obesity and type 2 diabetes [4–7]. This is usually in the presence of hyperglycaemia and dyslipidemia, which are known triggers of inflammatory and oxidative stress pathways. In cells and in animal models, it is becoming apparent that inflammatory cytokines and reactive oxygen species (ROS) contribute to the early development of insulin resistance [8–10].

Humans spend the majority of their time in the postprandial state, which is hypothesised to be both proinflammatory and pro-oxidative and may directly affect insulin resistance in skeletal muscle, contributing to development of type 2 diabetes [1, 2]. However, the available literature is conflicting as to whether circulating inflammatory cytokines change in response to meals, although a consensus is generally reached in respect to stimulation of postprandial IL6 [11–14]. Whether high-fat and high-carbohydrate meals differentially modulate the inflammatory or oxidative stress responses in humans is also debated [15, 16]. Therefore, the aim of this study was to examine circulating and skeletal muscle gene expression of inflammation and oxidative stress markers in the fasting state and after a physiological challenge by a high-carbohydrate or high-fat meal in healthy, non-obese, normoglycemic individuals, 7 of whom reported a family history of type 2 diabetes.

## 2. Methods

### 2.1. Study Population

The study population has been described previously [17] and consisted of 15 nonsmoking, nondiabetic normolipidemic subjects, aged 18–55 years who reported either no family history (FH−, *n* = 8) or a strong family history of type 2 diabetes (FH+, *n* = 7). Subjects were excluded if weight changed by ≥2 kg in the preceding 6 months, if they drank more than 2 standard alcoholic drinks per day, if they were taking any medications known to affect insulin-sensitivity or had a personal history of type 2 diabetes or cardiovascular disease. The study was approved by the Human Research Ethics Committee at St. Vincent's Hospital, Sydney, and each subject gave written consent prior to the start of the study.

### 2.2. Study Design and Procedures

A randomized, paired, crossover design was used. On the first visit, a hyperinsulinaemic-euglycaemic clamp (50 mU*·*m^−2^ *·*min^−1^) and a dual energy X-ray absorptiometry (DXA) scan (Lunar DPX-Lunar Radiation, Madison, WI) were performed to assess insulin-sensitivity and body composition, respectively. At visits 2 and 3, which were performed at least 3 days and no more than 2 weeks apart, a meal rich in either carbohydrate or fat was consumed after a prolonged (20 h) fast (randomly assigned) (Table 2). In the high-fat meal, 34% of fat was saturated, 29% polyunsaturated, and 38% monounsaturated. The subjects were instructed not to consume alcohol 24 h prior to every study day. Biopsies from musculus vastus lateralis were taken fasting and 3 h after meal. Blood samples for analysis of circulating inflammatory markers of interest were also assessed at baseline and 3 h after meal. Glucose, insulin, free fatty acid (FFA), and triglyceride concentrations were assessed at 0, 30, 60, 120, 180, and 240 minutes after completion of the meal.

### 2.3. Biochemical Analysis

Skeletal muscle expression of the following genes was studied: complement component C3 (*C3*), tissue-type plasminogen activator (*PLAT*), human forkhead-box C2 gene (*FOXC2*), nuclear factor kappa B (*NFKB1*), inhibitor of nuclear factor kappa B kinase beta subunit (*IKBKB*), hypoxia inducible factor 1, alpha subunit (*HIF1*α**), *IL6*, heme-oxygenase-1 (*HMOX1*), endothelial nitric oxide synthase (*NOS3*), heat shock 70 kDa protein (*HSPA1A*), Cu/Zn-superoxide dismutase (*SOD1*), and glutathione peroxidase (*GPX1*). Muscle RNA was extracted as described [18]. Briefly, cDNA was prepared from RNA by use of Superscript II and oligo dT primers (Invitrogen, VIC, Australia). Most primers were designed using MacVector (Sigma Aldrich, Castle Hill, NSW, Australia), except for *IL6*, *HMOX1*, *HSPA1A*, *NOS3*, *SOD1*, and *GPX1*, which were purchased as pre-made primer-probe sets from Applied Biosystems (NSW, Australia). Real-time quantitative PCR was performed with the 7900HT Fast Real-Time PCR System (Applied Biosystems, NSW, Australia) with Power SYBR Green PCR Master Mix or TaqMan Universal Master Mix, as appropriate (Applied Biosystems, NSW, Australia), according to the manufacturer's instructions. The Ct value for every sample was measured in duplicate and was normalized to beta actin (*ACTB*) expression, which was not different between groups at baseline and was not altered in response to either meal.

Circulating levels of C-reactive protein (CRP), serum amyloid A (SAA), and TNF*α* were determined in duplicate at baseline and 3 h, using LincoPlex multiplex immunoassay (Linco Research Inc., St Charles, MO), and the intra-assay CVs were 6%, 8%, and 7%, respectively. Serum IL6 and IL18 were determined using highly sensitive ELISA kits (R&D Systems Inc., MN, USA) and the intra-assay CVs were 8% and 7%, respectively. Insulin and total adiponectin concentrations were measured by RIA (Linco Research, St Charles, MO). Total antioxidant status (TAS) was analyzed by a colorimetric assay kit (Calbiochem, Merck, Germany) with an intra-assay CV of 7% and plasma Cu/Zn-SOD by an ELISA kit (Calbiochem, Merck, Germany) with an intra-assay CV of 5%.

### 2.4. Statistical Analysis

Data is presented as mean ± SEM. Statistics were analysed with StatView 5.0 (SAS Institute, Cary, NC). Basal differences between the groups were evaluated by student's *t-*test, and repeated-measures ANOVA was used to determine differences in response to the meal by group and time. Paired *t-*test analysis was performed to evaluate the effect of meal in the whole cohort when no group effect was observed. Correlations were performed using Pearson's correlation coefficient. Significance was set at *P* ≤ 0.05.

## 3. Results

### 3.1. Baseline and Postprandial Characteristics by FH Status

As we have previously reported in this cohort, individuals with (FH+) and without (FH−) a family history of diabetes had similar BMI, body fat composition by DXA, serum lipid profiles, and insulin sensitivity as assessed by glucose infusion rate necessary to maintain euglycemia during the final 30 minutes of the hyperinsulinemic clamp (Table 1). Fasting levels of insulin, glucose, and triglycerides were also not significantly different between FH+ and FH−, and there was no difference in the incremental areas under the curve for glucose, FFA, and triglyceride response to either the high-fat or high-carbohydrate meals. However, FH+ had a greater insulinemic response to both the high-carbohydrate and high-fat meal compared with FH− (Table 1, *P* < 0.02). Since we did not detect differences between groups at baseline or in response to meals for any markers of inflammation and oxidative stress, except for increased baseline HMOX1 gene expression in FH+ (6.0 ± 1.0 AU versus 2.7 ± 0.3 AU, *P* = 0.03), groups are combined for reporting by meal-type below.

### 3.2. High-Carbohydrate versus High-Fat Meal Response

**Figure 1:**
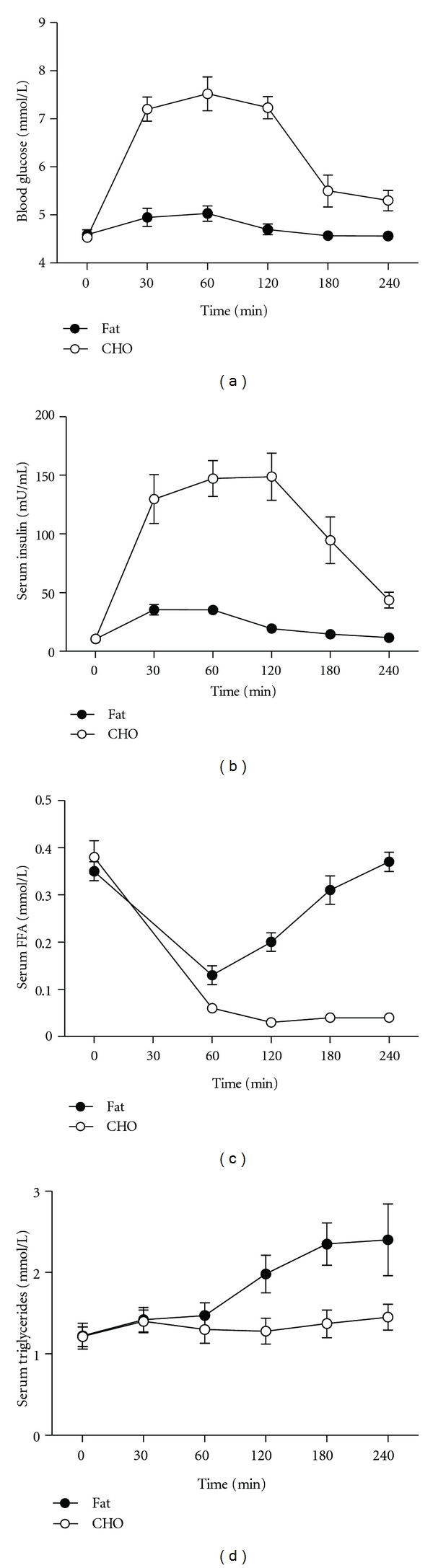
Levels of plasma (a) insulin, (b) glucose, (c) free fatty acid (FFA), and (d) triglycerides fasting and in response to high-carbohydrate or high-fat meals. Footnote: statistics were performed by repeated measure ANOVA.

**Figure 2:**
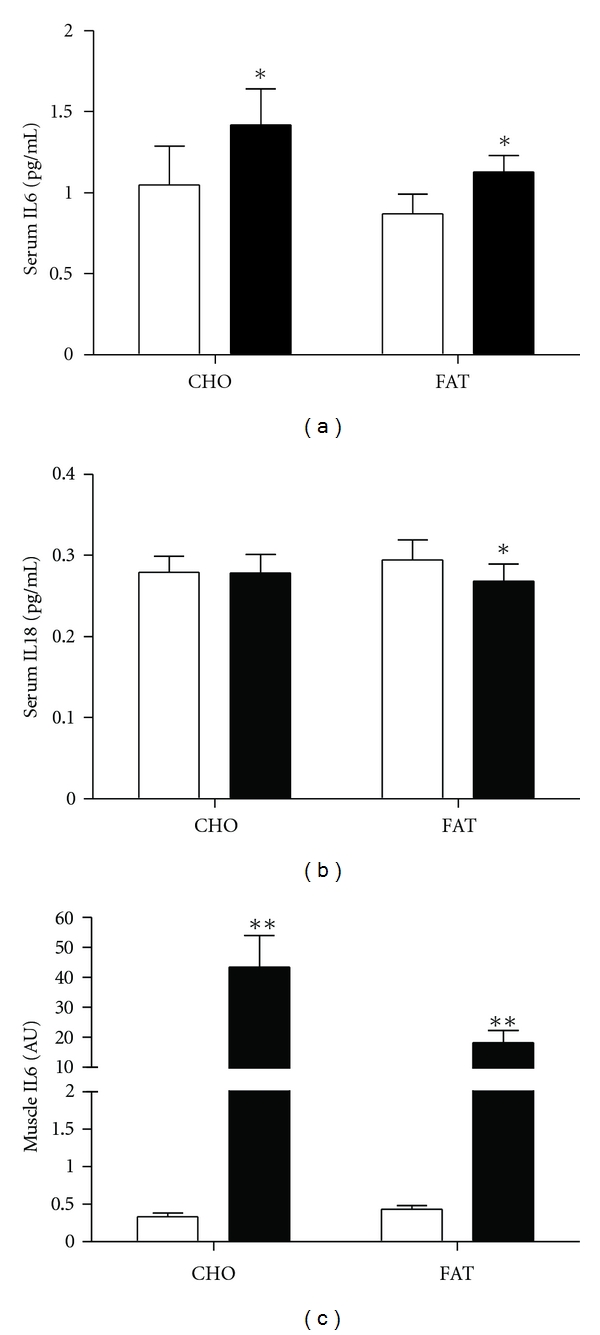
Circulating and muscle gene expression of inflammatory markers in the fasting state and 3 hours after either high-carbohydrate or high-fat meals. Footnote: statistics were performed by Student's *t-*test, *P < 0.05.

As expected, glucose and insulin responses were higher (Figures 1(a) and 1(b)) and FFA (Figure 1(c)) lower after the high-carbohydrate versus the high-fat meal and triglyceride concentration was raised more after the high-fat meal (Figure 1(d)). Plasma IL6 was elevated by 30–40% at 3 hours after each meal, independently of meal type (*P* < 0.05, Figure 2(a)). A positive relationship was observed between the change in circulating IL6 and change in insulin following the high-carbohydrate meal (*R* ^2^ = 0.28, *P* = 0.04). Circulating IL18 was decreased after the high-fat meal only (*P* < 0.01, Figure 2(b)). The systemic levels of CRP, TNF*α*, adiponectin, and serum amyloid A were not changed by either meal type (data not shown).

Skeletal muscle *IL6* expression increased by more than 1000-fold after each meal, without significant differences between meals (*P* < 0.005, Figure 2(c)). The other inflammation markers measured in skeletal muscle (*C3*, *FOXC2*, *IKBKB*, and *NFKB1) *were not changed with either meal (data not shown).

**Figure 3:**
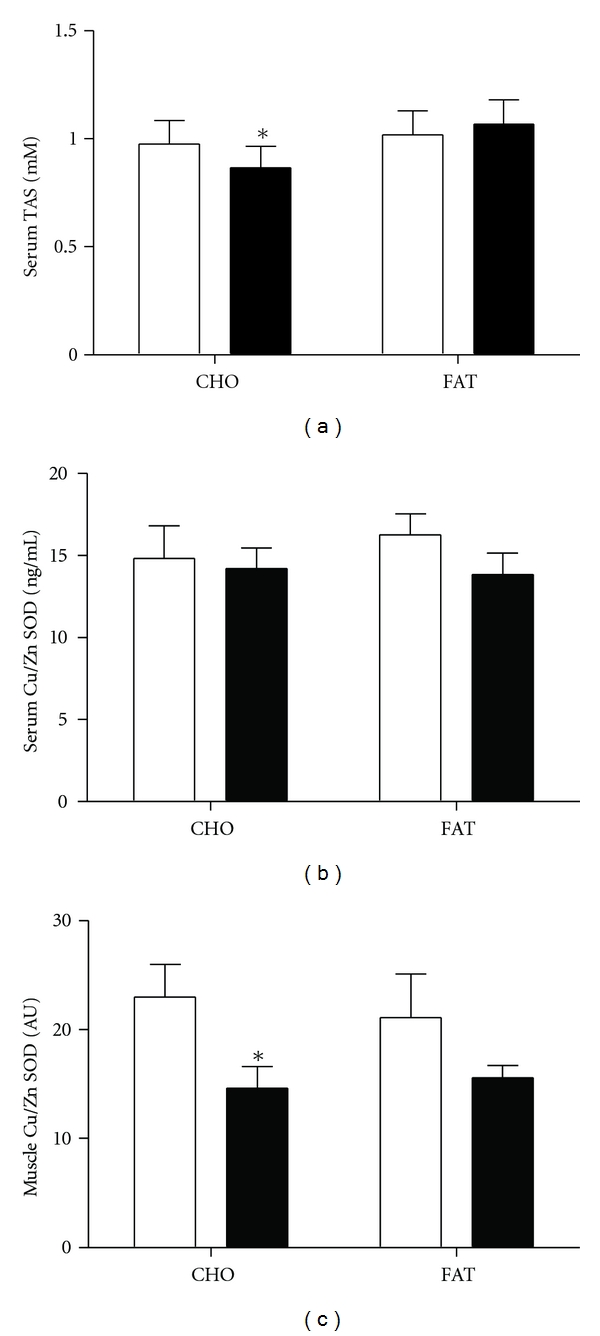
Fasting and 3-hour levels of (a) plasma total antioxidant status (TAS), (b) plasma Cu-Zn superoxide dismutase, and (c) Cu-Zn superoxide dismutase expression in skeletal muscle in response to high-carbohydrate or high-fat meals. Footnote: statistics were performed by paired *t*-test, *P ≤ 0.05, **P ≤ 0.01.

In response to the high-carbohydrate meal, total antioxidant status (TAS) decreased (*P* = 0.03, Figure 3(a)), without change in response to the high-fat meal. Plasma levels of the antioxidative enzyme Cu/Zn-SOD were unchanged (Figure 3(b)), although its expression in muscle decreased in response to the high-carbohydrate meal (*P* = 0.02, Figure 3(c)). The skeletal muscle expression of *HIF1*α*, HMOX1, NOS3*, *HSPA1A*, and *GPX1* did not change with either meal (data not shown).

### 3.3. Relationships between Insulin Sensitivity or Fatness, and Inflammation and Oxidative Stress

We observed relationships between CRP and BMI (*r* = 0.56, *P* = 0.03) and fasting levels of circulating IL6 also tended to correlate with BMI (*r* = 0.51, *P* = 0.06). However, no other relationships were observed for other inflammatory markers either in fasting state or after the meal. Of the oxidative stress markers examined, only muscle expression of *GPX1 *positively correlated with BMI (*r* = 0.4, *P* = 0.04).

## 4. Discussion

The aims of the present study were to (i) examine the inflammatory and oxidative stress response to meals in a healthy nondiabetic cohort and (ii) to compare responses between healthy normoglycemic individuals with and without a family history of type 2 diabetes. The study established that there was a substantial elevation in both circulating and muscle gene expression of IL6, independently of the type of meal consumed and without increases in any other measured inflammatory markers.

Evidence surrounding proinflammatory effects of meals is controversial. Some meal studies have reported a proinflammatory response, indicated by an increase in endotoxemia [19], activation of blood leukocytes [20], induction of nuclear proinflammatory transcription factors [21] and increased CRP [11]. In contrast, others observed no change in adiponectin, CRP, and C3 and a decrease in circulating TNF*α* [13, 14]. These discrepancies may be a result of differences in meal composition, energy content, and particularly the adiposity and disease status of the population studied. We confirm the most consistent finding amongst these reports, a postprandial increase in circulating IL6, and we confirm that this is independent of macronutrient composition of the meal [11, 14]. We also extend previous findings by showing that the increase in circulating IL6 was due, at least in part, to increased skeletal muscle expression, with a >1000-fold increase in mRNA detected in this tissue.

Chronic elevation of systemic IL6 in humans is associated with type 2 diabetes, obesity, and sedentary lifestyle and may be attributable to increased number of macrophages residing in the adipose tissue [22]. In contrast, exercise training causes an acute elevation in circulating IL6 released from muscle during contraction which has been reported to stimulate glucose uptake [23, 24]. Thus, the source of IL6, namely, adipose tissue versus skeletal muscle and the chronic versus pulsatile response in obese and/or diabetic versus healthy subjects may explain the controversies regarding the role of IL6 in human health and disease [25]. It is well recognised that obese- and insulin-resistant subjects with chronic low grade inflammation improve glycaemia in concert with a reduction in cytokines levels following interventions such as weight loss [22]. However, reducing IL6 levels, in the absence of adipose tissue inflammation may be ineffective. Since the muscle IL6 response was observed in all individuals, and in the absence of any increases in inflammatory markers, we speculate that the meal-related release of IL6 is physiological and may enhance glucose uptake. In support of this hypothesis, intravenous IL6 infusion during the hyperinsulinemic clamp increases glucose disposal in healthy individuals [26], similar to the effects of IL6 during exercise [23]. Interestingly, both continuous and intermittent administration of IL6 to rats increases insulin sensitivity via mitochondrial uncoupling and enhanced skeletal muscle fat oxidation capacity [27]. Moreover, the IL6 global knockout mouse model is insulin-resistant, whilst the IL6 transgenic mouse is protected from diet-induced obesity and insulin-resistance [28]. In this study, none of the variables examined were related to the increase in muscle IL-6 expression. However, we clarify that a positive relationship was observed between the increase in serum insulin and serum IL6 in response to the high-carbohydrate meal only. This may indicate that more insulin resistant subjects have greater IL6 responses. This result is in contrast to Dixon et al., who compared the serum IL6 response to a mixed meal in active and inactive men. In this study, the increase in serum IL-6 was not different between groups, despite significantly higher glucose and insulin responses in the sedentary men [29]. Together, we propose these findings support a beneficial physiological postprandial role of IL6 in healthy individuals.

IL18 is proinflammatory and levels are elevated in obesity and type 2 diabetes [30–32] and are correlated with traits of the metabolic syndrome [33]. Knockout of IL-18 or its receptor results in obesity and insulin resistance [34], and fasting IL-18 levels are reduced following weight loss in humans [35]. However, whether these levels are changed in response to a meal is unclear. In this study, the high-fat meal produced a small reduction in circulating IL18, but no changes in IL18 following the high-carbohydrate meal. This is in contrast to Esposito et al. [36], who observed increased IL18 after the high-fat meal and decreased IL18 after a high-carbohydrate meal in 60 individuals with and without type 2 diabetes. The reason for the discrepancies between studies is unclear. In vitro studies have shown that intermittent and sustained hyperglycaemic exposure of 3T3L1 adipocytes increases the secretion of IL18. This occurs in concert with increased ROS production, both of which were blocked with the antioxidant *N*-acetylcysteine suggesting that ROS generation may mediate hyperglycaemic elevations in IL18.

The postprandial phase has been described as pro-oxidative [15, 16, 37–39] with the magnitude of the oxidative stress response linked to the carbohydrate content of the meal and the level of hyperglycaemia and hyperinsulinemia evoked [16]. However, data gathered in obese humans has been controversial, with studies reporting either increased [35] or decreased [40] ROS emission from skeletal muscle mitochondria *ex vivo* in the postprandial state. In the present study, plasma TAS levels were decreased in response to the high-carbohydrate meal only, suggesting increased consumption of antioxidative molecules associated with hyperglycaemia-induced oxidative stress [16]. Muscle expression of Cu/Zn-*SOD* was also decreased in response to the high-carbohydrate meal, but the plasma levels were unchanged. In this study, we focussed on the antioxidative response to meals, rather than assessing markers of oxidative-stress-induced damage. Based on the findings observed here, we predict that markers of lipid peroxidation, including the thiobarbituric acid reactive substances (TBARS) or malondialdehyde (MDA) assays, would increase following a high-carbohydrate meal, but not following high-fat meals.

Individuals with a family history of type 2 diabetes have a 40% lifetime risk of developing T2D with a 20% risk of developing glucose intolerance (1) and thus are an excellent human model to detect factors that contribute to the early development of insulin resistance. Previously, in this cohort, we have reported metabolic inflexibility in response to a high-fat meal (2) and a blunted peptide YY (PYY) increment in response to a high-carbohydrate meal (3), both of which may contribute to later insulin resistance, weight gain, and eventual T2D. However, in this study, the basal and postprandial inflammatory and oxidative stress measures were not different between FH+ and FH−, with the exception of elevated basal expression of muscle heme-oxygenase-1 (HMOX1), which was increased in FH+. Therefore, it may be that differences in inflammatory and oxidative stress responses are acquired later in the life course of type 2 diabetes. However, due to the small group size caution should be exercised in making this interpretation.

The reports on HMOX1 in type 2 diabetes are controversial. Some studies reported increased circulating levels of plasma HMOX1 [41] and gene expression of HMOX1 in circulating monocytes [42] in type 2 diabetes, with reduction in some individuals after metabolic normalisation. However, other studies have reported reduced levels of HMOX1 in muscle and leukocytes at a late stage of type 2 diabetes patients [43]. Administration of the HMOX1 inducer, hemin, improves glucose metabolism in Zucker diabetic rats and streptozotocin rats (39), and HMOX1 upregulation improves fasting and postprandial glucose in humans [44]. Together, these data suggest that fasting muscle HMOX1 may be upregulated to protect against oxidative-stress-induced insulin resistance, a response that may be lost in type 2 diabetes.

In conclusion, macronutrient composition of the diet may differentially alter the postprandial pro-oxidative milieu, with high-carbohydrate meals potentially leading to greater oxidative stress response. However, both meals increased circulating IL6, regardless of the type of nutrient consumed. Our data suggests that the source of this increase was skeletal muscle, and since this was observed in absence of other inflammatory changes, we propose that IL6 release is part of healthy postprandial muscle metabolism, similar to that reported in the postexercise state.

## Figures and Tables

**Table 1:** Baseline characteristics of study participants with (FH+) and without (FH−) a family history of T2D.

	FH−	FH+
*N* (Male/Female)	7 (2/5)	8 (2/6)
Age (years)	41 ± 3	47 ± 2
Weight (kg)	75.5 ± 5.5	69.5 ± 3.3
BMI (kg/m^2^)	26.5 ± 2.1	26.0 ± 1.9
Total body fat (%)	36 ± 4	36 ± 4
Abdominal fat (kg)	2.5 ± 0.4	1.9 ± 0.3
Total cholesterol (mmol/L)	4.6 ± 0.3	5.1 ± 0.2
HDL cholesterol (mmol/L)	1.2 ± 0.08	1.3 ± 0.07
LDL cholesterol (mmol/L)	2.9 ± 0.2	3.2 ± 0.15
Triglycerides (mmol/L)	1.1 ± 0.2	1.3 ± 0.2
GIR (*μ*mol min^−1^/kg FFM)	78.7 ± 9.3	73.5 ± 9.2
Glucose iAUC (CHO)	429 ± 65	492 ± 55
Glucose iAUC (Fat)	43 ± 13	53 ± 16
Insulin iAUC (CHO)	15924 ± 3190	31081 ± 3993*
Insulin iAUC (Fat)	1895 ± 392	3379 ± 322*

Data are given mean ± SEM, GIR: Glucose infusion rate necessary to maintain euglycemia during the hyperinsulinemic clamp; AUC: area under the curve, *P < 0.02.

**Table 2:** Food items (grams) and the macronutrient compositions of the high-fat and carbohydrate meals.

Item	Protein	Fat	CHO
High-carbohydrate meal			

Weetbix cereal (60 g)	7.2	0.8	40
No fat milk (300 mL)	14.4	0.3	20.2
Spaghetti, tinned (1 cup)	5.1	3	34
Margarine (5 g)	0	4	0
Orange juice (25 mL)	1	0	25
Wholemeal bread (75 g)	7.5	1.3	33.5
Banana (150 g)	1	0	30

Total (g)	36.2	9.4	182.7
Energy (kJ)	605	353	3051
E%	15.1	8.8	76.1

High-fat meal			

Cheese (44 g)	11	15.4	0
Eggs (118 g)	14.2	11.8	0
Oil (20 mL)	3	20	0
White bread (30 g)	0	0.5	13
Tomato (100 g)	0	0	3
55% cream (30 mL)	0	16.5	0
Strawberries (100 g)	0	0	3
Almonds (30 g)	6	15	1

Total (g)	34.2	79.2	20
Energy (kJ)	571	2978	334
E%	14.7	76.7	8.6

## Abbreviations

ROS: Reactive oxygen species
PYY: Polypeptide Y
DXA: Dual energy X-ray absorptiometry
CRP: C-reactive protein
SAA: Serum amyloid A
TAS: Total antioxidant status
SOD: Superoxide dismutase
CHO: Carbohydrate.
